# Classifying and Evaluating Fetuses With Ventriculomegaly in Genetic Etiologic Studies

**DOI:** 10.3389/fgene.2021.682707

**Published:** 2021-07-02

**Authors:** Meiying Cai, Hailong Huang, Liangpu Xu, Na Lin

**Affiliations:** Medical Genetic Diagnosis and Therapy Center, Fujian Maternity and Child Health Hospital, Affiliated Hospital of Fujian Medical University, Fujian Key Laboratory for Prenatal Diagnosis and Birth Defect, Fuzhou, China

**Keywords:** ventriculomegaly, copy number variation, SNP-array, fetal screening, genetic evaluation

## Abstract

The association between genetics and fetuses with ventriculomegaly (VM) is unknown. This study aimed to classify and evaluate abnormal copy number variations (CNVs) in fetuses with VM. From December 2016 to September 2020, amniotic fluid or umbilical cord blood from 293 pregnant women carrying fetuses with VM was extracted for single-nucleotide polymorphism microarray (SNP array). Among 293 fetuses with VM, 31 were detected with abnormal CNVs, including 22 with pathogenic CNVs (7.51%) and nine with variation of uncertain clinical significance (VUS) CNVs (3.07%). Of the 22 fetuses with pathogenic CNVs, 13 had known disease syndromes. Among the 293 fetuses, 133 had mild isolated VM [pathogenic CNVs, 7/133 (5.26%)]; 142 had mild non-isolated VM [pathogenic CNVs, 13/142 (9.15%)]; 12 had severe isolated VM [pathogenic CNVs, 2/12 (16.67%)]; and six had severe non-isolated VM (no abnormal CNVs was detected). There was no statistical significance in the rate of pathogenic CNVs among the four groups (*P* = 0.326, *P* > 0.05). Among the 267 fetuses with successful follow-up, 38 were terminated (of these, 21 had pathogenic CNVs). Of the 229 fetuses, two had developmental delay and the remaining 227 had a good prognosis after birth. Overall, the results are useful for the detection of fetal microdeletion/microduplication syndrome and for the accurate assessment of fetal prognosis in prenatal consultation.

## Introduction

Ventriculomegaly (VM) is the most common abnormality observed on prenatal ultrasound (Salomon et al., 2010), and the reported incidence of VM is 0.03 to 2.20% (D’Addario et al., 2007). According to the degree of broadening, VM is generally divided into two categories: mild (10–15 mm) and severe (≥15 mm) (Bloom and Bloom, 1998). The etiology of VM is relatively complex; this could be a normal phenotype or central nervous system abnormalities, such as abnormal development of the brain line structure or local space occupancy. It may also be a result of chromosomal abnormalities and virus infection, among others (Kelly et al., 2001; Weichert et al., 2010; Pagani et al., 2015).

Evaluation of the width of VM has become a routine prenatal ultrasonography (Griffiths et al., 2017). The width of VM is a significant prognostic factor in determining the outcome of fetal VM (Gezer et al., 2016). About one-third of the fetuses with mild VM can recover and normalize spontaneously during pregnancy (Wax et al., 2003). However, severe VM is often associated with chromosomal abnormalities, especially trisomy 21 syndrome and chromosomal microdeletion/microduplication, which can lead to unfavorable pregnancy outcomes (Letouzey et al., 2017).

The traditional karyotype analysis has become the gold standard for the clinical detection of chromosome abnormalities due to its ability to detect chromosome number abnormalities and chromosomal structure abnormalities of large segments. Single-nucleotide polymorphism array (SNP array) has a higher resolution and can detect copy number variations (CNVs) that karyotype analysis cannot (Wapner et al., 2012). In recent years, SNP array has been widely used in chromosomal screening for postnatal, prenatal, and recurrent abortion cases (Sahoo et al., 2017; Wang et al., 2018). However, there have been few reports on the association between VM and CNVs in fetuses (Kennelly et al., 2010; Shaffer et al., 2012; Donnelly et al., 2014; Wu and Sun, 2015; Hu et al., 2017; Letouzey et al., 2017; Ron et al., 2017). In this study, SNP array was used to evaluate the CNVs of VM in fetuses and to explore the value of SNP array in prenatal diagnosis of VM.

## Materials and Methods

### Clinical Data

**FIGURE 1:**
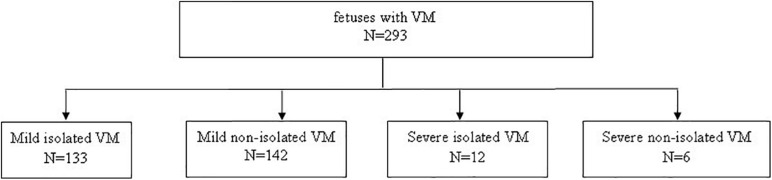
Enrollment of fetuses.

A total of 293 pregnant women carrying fetuses with VM from December 2016 to September 2020 at Fujian Maternal and Child Health Hospital were selected as the research subjects. The age of pregnant women ranged from 18 to 48 years, and the gestational age ranged from 16^+3^ to 37^+5^ weeks. The inclusion criterion for this study was fetus with VM. The exclusion criterion was a twin pregnancy. According to the degree of VM and whether they were combined with other ultrasound abnormalities, the 293 cases were divided into four groups (Figure 1): (1) 133 cases with mild isolated VM (only VM < 15 mm); (2) 142 cases with mild non-isolated VM (VM < 15 mm combined with other ultrasound abnormalities); (3) 12 cases with severe isolated VM (only VM ≥ 15 mm); and (4) six cases with severe non-isolated VM (VM ≥ 15 mm combined with other ultrasound abnormalities). The demographic characteristics for all four groups are shown in Table 1. This study was approved by the Ethics Committee of Fujian Maternal and Child Health Hospital, and the subjects signed the informed consent forms.

**TABLE 1:** The demographic characteristics for all four groups.

Group	Total (*n*)	Mother’s age (years)	Gestational age (ages)	Cord blood (*n*)	Amniotic fluid (*n*)	Pregnancy outcome		
						Loss to follow-up (*n*)	TD (*n*)	TP (*n*)
Mild isolated VM	133	18 to 48	16^+3^ to 34^+2^	85	48	16	95	22
Mild non-isolated VM	142	18 to 45	17^+3^ to 37^+5^	98	44	7	129	6
Severe isolated VM	12	20 to 44	16^+5^ to 32^+5^	6	6	2	3	7
Severe non-isolated VM	6	22 to 41	20^+3^ to 33^+2^	4	2	1	2	3

*TD, term delivery; TP, termination of pregnancy; VM, ventriculomegaly.*

### SNP-Array Analysis

Methods published previously were applied (Cai et al., 2020). After routine disinfection, 20 ml of amniotic fluid or 2 ml of umbilical cord blood was extracted by ultrasound-guided transabdominal puncture for SNP array. Genomic DNA was extracted from the samples according to the instructions of the Qiagen DNA Blood Mini Kit. Genomic DNA was digested, ligated, amplified, purified, segmented, labeled, hybridized, and scanned according to the standard operating procedures of on the Affymetrix SNP Array CytoScan 750K (Affymetrix, Santa Clara, CA, United States). The data obtained from the scan were analyzed by the software Chromosome Analysis Suite (ChAS) version 3.2 (Affymetrix, Santa Clara, CA, United States). The test results are annotated based on GRCh37 (hg 19). Through comparison and analysis of public databases such as International Standards for Cytogenomic Arrays (ISCA), National Center for Biotechnology Information (NCBI), Decipher Database, and Online Mendelian Inheritance in Man (OMIM), the nature of CNVs detected was determined. The nature of CNVs was divided into five categories (Hanemaaijer et al., 2012): pathogenic CNVs, likely pathogenic CNVs, a variation of uncertain clinical significance (VUS) CNVs, likely benign CNVs, and benign CNVs.

### Statistical Analysis

SPSS 22.0 software (SPSS, Inc., Chicago, IL, United States) was used to process the data. The chi-square test was used for comparison of the rate of pathogenic CNVs in different types of VM fetuses, and *P* < 0.05 was considered statistically significant.

### Follow-Up

All patients were followed up by telephone. The pregnancy outcome, postpartum growth, and development information were recorded.

## Results

### Abnormal CNVs in the Fetus With VM

Among 293 fetuses with VM, SNP array detected 31 cases with abnormal CNVs, including 22 cases with pathogenic CNVs (7.51%, 22/293) and nine cases with VUS CNVs (3.07%, 9/293). Of the 22 cases of pathogenic CNVs, 13 cases had known disease syndromes, including four cases of Down syndrome, three cases of 16p11.2 deletion syndrome, and one case each of Edwards’ syndrome, 17q12 duplication syndrome, Miller–Dieker syndrome, Sotos syndrome, Wolf–Hirschhorn, and 1p36 deletion syndrome. In the remaining nine cases with pathogenic CNVs, the SNP array identified deletions of 15q11.2q13.1, 5p15.33p13.3, 21q11.2q22.11, 16p13.3, 16p13.11, and 1p36.33p36.32, and duplications of 12p13.33p11.1, 16p13.11, and 7q36.3.

Aside from the 13 cases whose parents refused verification, SNP-array verification was performed on the parents of nine fetuses with abnormal CNVs. *De novo* CNVs were found in seven fetuses, one fetus was found to have a 1.3-MB duplication in the 16p13.11 region originating from a paternal mutation, and one case had a 1.5-Mb duplication on chromosome 17 originating from a maternal mutation (Table 2).

**TABLE 2:** The pathogenic copy number variation in VM fetuses.

Case	SNP array	Size (Mb)	Severity of VM	Extra VM defect	Interpretation	Obstetrical outcomes	Inheritance
1	Chr21: x3	–	Mild	–	Down syndrome	TP	–
2	Chr21: x3	–	Mild	–	Down syndrome	TP	–
3	Chr21: x3	–	Mild	Nuchal translucency thickness, the alteration of wave A in the ductus venosus	Down syndrome	TP	–
4	Chr21: x3	–	Mild	Absent nasal bone	Down syndrome	TP	–
5	Chr18: x3	–	Mild	Echogenic intracardiac focus	Edwards’ syndrome	TP	–
6	Chr16: 29,567,296–30,190,029	0.6	Mild	–	Loss 16p11.2 (16p11.2 deletion syndrome)	TP	*De novo*
7	Chr16: 29,591,326–30,176,508	0.6	Severe	–	Loss 16p11.2 (16p11.2 deletion syndrome)	TP	–
8	Chr16: 28,810,324–29,032,280	0.2	Mild	Hyperechogenic bowel, echogenic intracardiac focus	Loss 16p11.2 (16p11.2 deletion syndrome)	TP	*De novo*
9	Chr17: 525–5,204,373	5.2	Mild	Bipedal varus, cerebellar primordial dysplasia, excessive amniotic fluid, and small gastric vesicles	Loss 17p13.3p13.2 (Miller–Dieker)	TP	–
10	Chr4: 112,192,577–127,874,789	15.6	Mild	Ventricular septal defect	Loss 4q25q28.1 (Wolf–Hirschhorn)	TP	–
11	Chr1: 246,015,892–249,224,684	3.2	Mild	–	Loss 1p36.33p36.23 (1p36 deletion syndrome)	TP	–
12	Chr17: 34,822,465–36,378,678	1.5	Mild	Hyaline septum dysplasia	Gain 17q12 (17q12 duplication syndrome)	TP	Maternal
13	Chr5: 175,416,095–177,482,506	2.0	Mild	Polyhydramnios	Loss 5q35.2q35.3 (Sotos syndrome)	TP	*De novo*
14	Chr12: 173,786–34,835,641	34.6	Mild	–	Gain12p13.33p11.1	TP	–
15	Chr15: 23,290,787–28,540,345	51.3	Mild		Loss 15q11.2q13.1	TP	–
16	Chr16: 15,058,820–16,309,046	1.3	Mild	–	Gain 16p13.11	TD	Paternal
17	Chr7: 155,347,675–156,348,660	1.0	Severe	–	Gain7q36.3	TP	*De novo*
18	Chr5: 113,576–32,785,953	31.8	Mild	Cerebellar dysplasia	Loss 5p15.33p13.3	TP	–
19	Chr21: 15,478,958–34,591,567	19	Mild	Ventricular septal defect	Loss 21q11.2q22.11	TP	–
20	Chr16: 85,880–536,631	0.4	Mild	FGR, ventricular septal defect, persistent left superior vena cava	Loss 16p13.3	TP	*De novo*
21	Chr16: 15,422,960–16,508,123	1.0	Mild	Echogenic intracardiac focus	Loss 16p13.11	TP	*De novo*
22	Chr1: 849,466–4,894,800	4.0	Mild	Bilateral renal enlargement	Loss 5p15.33p13.3	TP	*De novo*

*FGR, fetal growth restriction; TD, term delivery; TP, termination of pregnancy; VM, ventriculomegaly.*

Among the fetuses with VM, nine cases had VUS CNVs, among which eight cases had chromosomal microdeletion or microduplication involving segment sizes of 0.4–2.5 Mb (Table 3).

**TABLE 3:** The variants of uncertain clinical significance in VM fetuses.

Case	SNP array	Size (Mb)	Severity of VM	Extra VM defect	Interpretation	Obstetrical outcomes	Inheritance
1	Chr1: 145,375,770–145,770,627	0.7	Mild	–	Loss 1q21.1	TD	*De novo*
2	Chr3: 42,875,130–43,309,436	0.4	Mild	–	Loss 3p22.1	TD	–
3	Chr3: 12,183,082–12,669,247	0.5	Mild	–	Gain 3p25.2	TD	–
4	Chr3: 1,855,754–2,663,625	0.8	Mild	–	Loss 3p26.3	TD	–
5	Chr16: 75,275,963–76–432–398	1.2	Mild	–	Gain 16q23.1	TD	–
6	Chr14: 46,782,405–49,288,860	2.5	Severe	–	Loss 14q21.2q21.3	TP	–
7	Chr15: 31,999,631–32,444,043	0.4	Severe	–	Gain 15q13.3	TP	*De novo*
8	Chr11: 20,745,930–21,780,075	1.0	Mild	Ventricular septal defect, uronephrosis	Gain 11p15.1p14.3	TP	–
9	Chr3,5,6,12,17,21: 163,256,369–197,791,601, 41,029,13746,313,469, 143,341,406161,527,784, 56,011,10077,134,151, 39,639,602–45,479,706, 28,124,165–42,352,287	99.1	Mild	–	Lack of heterozygosity 3q26.1q29, 5p13.1p11, 6q24.2q26, 12q13.2q21.2, 17q21.2q21.32, 21q21.3q22.2	TD	–

*TD, term delivery; TP, termination of pregnancy; VM, ventriculomegaly.*

### Comparison Pathogenic CNVs in VM Fetuses With or Without Other Ultrasound Abnormalities

**FIGURE 2:**
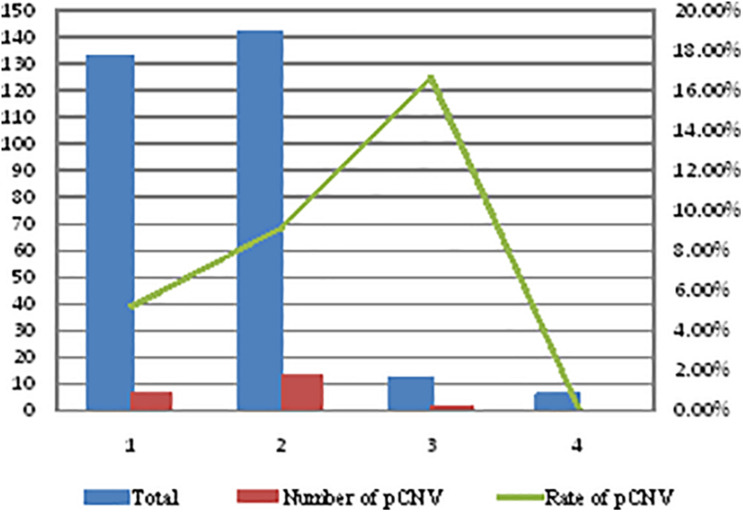
Comparison of the rate of pathogenic CNVs in four types of VM fetuses. 1, mild isolated VM group; 2, mild non-isolated VM group; 3, severe isolated VM group; 4, severe non-isolated VM group; VM, ventricular widening; CNVs, copy number variations; pCNV, pathogenic copy number variations.

The rate of pathogenic CNVs in four groups (mild isolated VM group, mild non-isolated VM group, severe isolated VM group, and severe non-isolated VM group) was different (Figure 2). Severe isolated VM and mild non-isolated VM groups had a higher rate of pathogenic CNVs than mild non-isolated VM and severe non-isolated VM groups, but there was no statistical significance in the rate of pathogenic CNVs among the four groups (*P* = 0.326, *P* > 0.05) (Figure 2).

### Pregnancy Outcomes and Follow-Up

Follow-up was performed in 267 cases, but were not completed in 26 cases. Among the 267 fetuses with follow-up, 38 fetuses were terminated, among which 21 were found with pathogenic CNVs, three were found with VUS CNVs, 10 were found with no abnormal CNVs but severe VM, and four were found with other severe malformations. Of the 229 cases with term delivery, two cases had developmental delay and the remaining 227 cases had a good prognosis after birth. Of the 22 fetuses with pathogenic CNV detected in the study, 21 fetuses were terminated. One fetus had a 1.3-Mb duplication in the 16p13.11 region. Ultrasound of the fetus only indicated the VM. It has been reported that such patients have great differences in clinical phenotypes, including developmental delay, learning difficulties, language disorders, and behavioral abnormalities, while some patients have no abnormal phenotypes (Ramalingam et al., 2011; Allach et al., 2020).

## Discussion

Evaluation of VM in the fetus has been internationally recommended as a routine part of the prenatal ultrasound (Paladini et al., 2007). In the past few decades, traditional karyotype analysis has been the gold standard. The Society of Obstetricians and Gynaecologists of Canada (SOGC) recommends amniocentesis for karyotype analysis and evaluation of congenital infection in mild to severe VM fetuses with or without other sonographic abnormalities (Hof and Wilson, 2005). In this study, SNP array was used to analyze 293 fetuses with VM to determine the underlying abnormal CNVs.

It was found that the incidence of pathogenic CNVs in non-isolated fetuses with VM (6.6–37.9%) was higher than that in isolated fetuses with VM (4.0–9.5%), and the incidence of pathogenic CNVs was not related to the degree of severity of VM (Shaffer et al., 2012; Donnelly et al., 2014). In this study, the rate of pathogenic CNVs in fetuses with mild isolated VM was 5.26%, 9.15% in the fetuses with mild non-isolated VM, and 16.67% in fetuses with severe isolated VM. The rate of pathogenic CNVs in fetuses with severe non-isolated VM was zero, which may be related to the small number cases (only six cases). There was no statistical significance in the rate of pathogenic CNVs among the four groups. Other studies have demonstrated similar results. For example, Shaffer et al. (2012) found that the rate of pathogenic CNVs in fetuses with isolated VM and non-isolated VM was 4.0 and 6.6%, respectively; Donnelly et al. (2014) reported that the rate of CNVs in fetuses with isolated VM and non-isolated VM was 8.7 and 17.2%, respectively; Wadt et al. (2012) reported on two familial submicroscopic terminal 6q deletions in fetuses with isolated VM; Fu et al. (2014) found MECP2 microduplication in four fetuses with VM; Xue et al. (2021) reported that detections of clinical significant CNVs were higher in non-isolated VM than in isolated VM (16.81% vs. 10.7%, *P* = 0.19). In the future, large-scale studies are required to determine the relationship between the incidence of genomic abnormalities and the severity of VM in fetuses. However, as fetuses with VM, regardless of their mild or severe, isolated, or non-isolated grouping, have a risk of pathogenic CNV, SNP array is recommended to exclude genomic abnormalities.

Among 293 fetuses with VM, SNP array detected 22 cases with pathogenic CNVs (7.51%). Previous studies have also shown that fetuses with VM are associated with CNVs (Wadt et al., 2012; Fu et al., 2014). Among the 22 cases with pathogenic CNVs, 13 cases were known pathogenic syndromes, among which Down syndrome was the most common (four cases). The number of Down syndrome cases was consistent with the study by Mckechnie et al. (2012). In this study, three cases of 16p11.2 deletion syndrome were detected, and the ultrasonic manifestations showed that two cases were mild VM and one case was severe VM. Two cases with 16p11.2 (proximal, BP4–BP5) and clinical features associated with 16p11.2 (TBX6) proximal region deletion may include developmental delay, cognitive impairment, language delay, autism spectrum disorder, neurologic issues including seizures, or electroencephalogram abnormalities. Incomplete penetrance has been observed; the penetrance has been reported at about 46.8%. One case has 16p11.2 (distal, BP2-BP3), SH2B1, a critical gene which has been reported in association with a variable and incompletely penetrant phenotype that may include developmental delay, obesity, behavioral problems, schizophrenia, and craniofacial dysmorphism (Hanson et al., 2014; Dell’Edera et al., 2018). VM may be a manifestation of this neurological susceptibility site. When the brain has severe VM, the prognosis of the fetus may be poor.

17q12 duplication syndrome was detected in one fetus where the size of the repeating fragment was about 1.5 Mb and contained 17 OMIM genes, which were inherited from the mother with a normal phenotype. In addition to VM, there was also hyaline septum dysplasia in this fetus. HNF1B is a key gene in this region that leads to abnormal phenotypes. It has been reported that the gene HNF1B is associated with a variable clinical presentation that includes developmental delay, behavioral problems, microcephaly, epilepsy, brain abnormalities, urinary malformation, and other abnormalities (Hardies et al., 2013; Kamath et al., 2018). Approximately 90% of cases with 17q12 duplication syndrome are inherited from parents with normal or slightly abnormal phenotypes. Parents without the phenotype cannot be sure whether the fetus is abnormal. Ultrasound indicates that VM is abnormal, which is usually considered to be related to the CNV.

Miller–Dieker syndrome was detected in one fetus with a deletion of copy number on chromosome 17 in the p13.3p13.2 region. The fragment was about 5.2 Mb in size and contained 77 OMIM genes. In addition to bilateral VM, ultrasound of the fetus with Miller–Dieker syndrome also showed bipedal varus, cerebellar primordial dysplasia, excessive amniotic fluid, and small gastric vesicles. It has been reported that patients with Miller–Dieker syndrome are characterized by no gyrus, more severe abnormalities in the posterior gyrus, developmental delay and epilepsy, congenital heart malformation, omphalocele, and joint contracture, among other symptoms (Hsieh et al., 2013; Proske and Beissert, 2015). Sotos syndrome was detected in one fetus with a deletion of copy number on chromosome 5 in the q35.2q35.3 region. The fragment was about 2.0 Mb in size and contained 49 OMIM genes. Ultrasound of the fetus showed polyhydramnios in addition to bilateral VM. It has been reported that the main clinical symptoms of patients with Sotos syndrome are special facial features, intellectual disability, and megacranial deformity (Baujat and Cormier-Daire, 2007; Panigrahi and Chaudhry, 2020).

1p36 microdeletion syndrome was detected in one fetus with a deletion of copy number on chromosome 1 in the p36.33p36.23 region. The fragment was about 7.7 Mb in size and contained 136 OMIM genes. Ultrasound of the fetus showed only VM. It has been reported that the main symptoms of patients with 1p36 microdeletion syndrome could manifest as cranial and facial abnormalities, intellectual disability, heart malformation, low muscle tone, developmental delay, and other symptoms (Gómez et al., 2011; Zhang et al., 2018). Wolf–Hirschhorn syndrome was detected in one fetus with a deletion of copy number on chromosome 4 in the q25q28.1 region. The fragment was about 15.6 Mb in size and contained 40 OMIM genes. Ultrasound of the fetus showed ventricular septal defect in addition to bilateral VM. It has been reported that Wolf–Hirschhorn syndrome can be characterized by a variety of congenital abnormalities, including developmental delay, intellectual disability, poor language ability, autistic tendencies, abnormal muscle strength, abnormal facial features, and deformities of the heart and nervous system (Akhtar, 2008; Blanco-Lago et al., 2013).

Single-nucleotide polymorphism array can not only detect subtle chromosomal structural abnormalities such as chromosomal microdeletions and microduplications but also detect VUS. It is relatively difficult to interpret the data, which brings great difficulties and challenges for genetic counseling. Many related studies reported that VUS cases accounted for less than 5% of all detected cases (Shaffer et al., 2012; Papoulidis et al., 2015). In this study, a total of nine cases of VUS were detected in 293 cases, and the detection rate was 3.07%, which was consistent with the data reported in the literature (Papoulidis et al., 2015). Although the genes included in these nine cases with VUS did not clearly lead to clinical phenotypes, it could not be completely ruled out that VUS was not related to the phenotypes of these cases, and further studies are needed to clarify its clinical significance. In recent years, next-generation sequencing (NGS) has been used to detect single-gene mutations and CNVs. Recent studies have shown that AIFM1 gene mutation which related to VM at early gestation (Berger et al., 2011) would lead to combined oxidative phosphorylation deficiency. More single-gene research will be reported in the future, which may provide a more comprehensive prenatal genetic diagnosis for fetuses with VM and better assess their future prognosis.

Single-nucleotide polymorphism array has been recommended as the first choice for detecting chromosomal CNVs, and it allows for detecting uniparental disomy (UPD), loss of heterozygosity (LOH), and low-level mosaic aneuploidies. CNV-seq technology based on NGS is a newly developed method for genome-wide CNV detection, which is characterized by its higher throughput, higher resolution, and lower cost than the SNP-array platform; the only problem is that it cannot detect UPD. With the advancement and reduced cost of NGS, the use of NGS as a detection method may be the trend in the future.

Single-nucleotide polymorphism array can sensitively detect the abnormal copy number of chromosomes in the genome in fetuses with VM, especially microdeletion/microduplication syndromes. This study showed that there was a risk of pathogenic CNVs in both mild and severe, isolated, and non-isolated cases. Therefore, SNP array is recommended for all fetuses with VM, which is more conducive to the accurate evaluation of fetal prognosis in prenatal clinical consultation.

## Data Availability Statement

The datasets presented in this study can be found in online repositories. The names of the repository/repositories and accession number(s) can be found below: https://www.ncbi.nlm.nih.gov/geo/, GSE163799.

## Ethics Statement

The protocol of this study was reviewed and approved by the Ethics Committee at the Fujian Provincial Maternal and Child Health Hospital (2014-042). The patients/participants provided their written informed consent to participate in this study.

## Author Contributions

LX: study design. NL: literature search and data analysis. MC: experiment performance. MC and HH: data interpretation and writing. All authors contributed to the article and approved the submitted version.

## Conflict of Interest

The authors declare that the research was conducted in the absence of any commercial or financial relationships that could be construed as a potential conflict of interest.
